# Comparative study of ERP habituation to tones and fearful vocalizations in autism spectrum disorders: a translational biomarker for sensory hypersensitivity

**DOI:** 10.1038/s41380-025-03335-z

**Published:** 2025-11-05

**Authors:** Kohei Shimono, Natsuko Kashida, Kantaro Nishigori, Tsuyoshi Iwasaki, Ryo Mizui, Kazuhiko Yamamuro, Rio Ishida, Michihiro Toritsuka, Tsutomu Takeda, Hiroto Tanakoshi, Hidetaka Nagata, Nakao Iwata, Manabu Makinodan

**Affiliations:** 1 Research and Development, Sumitomo Pharma Co., Ltd., Osaka, Japan; 2 Department of Psychiatry, Nara Medical University School of Medicine, Kashihara, Nara Japan; 3 Center for Health Control, Nara Medical University, Kashihara, Nara Japan; 4 Department of Psychiatry, Fujita Health University School of Medicine, Toyoake, Aichi Japan; 5 Division of Transformative Psychiatry and Synergistic Research, International Center for Brain Science, Fujita Health University, Toyoake, Aichi Japan; 6 Department of Neuropsychiatry, Kumamoto University, Kumamoto, Japan

**Keywords:** Autism spectrum disorders, Physiology

## Abstract

Sensory issues are common in autism spectrum disorders (ASD) and can significantly affect daily living. The phenomena of gating and habituation of event-related potentials (ERPs) to repetitive stimuli have been suggested as potential biomarkers reflecting atypical sensory processing in ASD. Sensory hypersensitivity and anxiety are closely related in ASD, and habituation to emotionally evocative stimuli may serve as a more sensitive biomarker for sensory hypersensitivity symptoms. However, previous studies have primarily used tonal stimuli, and there has been little investigation into whether habituation to emotionally evocative sounds is impaired in ASD patients. In this study, we compared the degree of habituation of the P1-N1 peak-to-peak amplitude in response to repeated tones and fearful vocalizations between control and ASD groups. Contrary to expectations, no significant difference was observed for fearful vocalizations between the groups, while ASD patients showed significantly reduced habituation to tonal sounds in the left parieto-occipital region. Furthermore, we found a significant correlation between the degree of habituation to tonal sounds in the left parieto-occipital region and sensory hypersensitivity symptoms in ASD patients, and similar abnormalities in BTBR mice, an animal model of ASD. These results suggest that habituation to tonal sounds, rather than emotionally evocative stimuli, may serve as a translational biomarker reflecting sensory hypersensitivity symptoms.

## Introduction

Autism spectrum disorders (ASD) are a neurodevelopmental condition characterized by deficits in social communication and interaction and in restricted and repetitive behaviors. Over the years, the emphasis in research on ASD has mainly been on social and communication challenges. However, in recent years, there has been an increasing focus on sensory issues, as recognized by the latest diagnostic criteria such as the Diagnostic and Statistical Manual of Mental Disorders, Fifth Revision (DSM-5) and International Classification of Diseases 11^th^ edition [1, 2]. These criteria now include sensory hyperresponsivity and hyposensitivity as part of the restricted and repetitive behavior domain. It has also been suggested that sensory processing difficulties are associated with various symptoms such as anxiety, restricted and repetitive behavior, and social difficulties, and they are believed to affect the quality of life of individuals with ASD and their families [3–6]. However, there are currently no effective treatments for sensory issues in ASD, highlighting the need for the development of new therapeutic drugs.

Abnormalities in sensory filtering functions, such as gating and habituation, have been suggested as potential causes of sensory hypersensitivity in ASD, based on studies using various neurophysiological measures. For example, previous studies have reported that individuals with ASD exhibit impaired sensory gating, as indicated by reduced attenuation of P1 and N1 component amplitudes in ERP responses to paired identical auditory stimuli [7, 8]. In addition, reduced habituation to repeated auditory stimuli has been reported, as reflected by a diminished decrease in the amplitudes of ERP components such as P1, N1, and P2 across successive stimulus presentations [9–16]. Within the autism ERP literature, the most frequently used nonlinguistic auditory stimuli are pure tones and broadband clicks, with the latter primarily being used to assess sensory gating. Additionally, decreased habituation of functional magnetic resonance imaging (fMRI) responses in the auditory cortex, as well as galvanic skin response (GSR) and magneto-encephalography (MEG) responses to repeated auditory stimuli, has also been reported [17, 18]. Furthermore, some studies have reported a correlation between these abnormalities in gating and habituation and the severity of sensory hypersensitivity symptoms, suggesting that these abnormalities could serve as biomarkers reflecting sensory hypersensitivity symptoms [8, 12, 14, 15].

In ASD, sensory hypersensitivity and anxiety are closely related, with some theories suggesting that it arises as a secondary effect of anxiety [19]. Indeed, several studies have reported that the degree of habituation decreases in response to stimuli that accompany negative emotions such as anxiety and fear [20–22], suggesting that habituation to emotionally evocative sounds may serve as a more sensitive biomarker for sensory hypersensitivity symptoms. However, previous studies have primarily utilized simple auditory stimuli such as tones and clicks, and there has been insufficient investigation into whether habituation and gating in response to emotionally evocative stimuli are altered in patients with ASD.

Psychiatric trials have some of the lowest success rates across therapeutic areas [23]. There are three reasons for low clinical trial success rates in psychiatry: diagnostic heterogeneity, endpoint subjectivity, and high placebo response rates [24]. There is a need to establish translational biomarkers that are objective and consistently quantifiable in both clinical and preclinical settings. Although ERP gating and habituation are expected to be potential clinical biomarkers for sensory hypersensitivity symptoms in ASD, most translational studies have utilized monogenic models of ASD such as Fmr1 KO mice to investigate sensory gating and habituation [25, 26]. In contrast, studies employing idiopathic autism models like BTBR mice remain relatively scarce.

In this study, we hypothesized that individuals with ASD exhibit impaired habituation to emotionally evocative stimuli, more so than to simple stimuli like tones. To test this hypothesis, we compared the degree of habituation of the P1-N1 peak-to-peak amplitude to tones and fearful vocalizations between the control and ASD groups using the same paradigm on the same subjects. Additionally, to determine whether the P1-N1 habituation to tones in the left parieto-occipital region of interest (ROI), where significant group differences were observed, could serve as a specific biomarker for sensory hypersensitivity symptoms, we examined correlations with various clinical symptom scores, including sensory hypersensitivity. Furthermore, to explore the potential of P1-N1 habituation to tones as a translational biomarker for drug development, we investigated whether similar P1-N1 habituation impairments observed in ASD patients could also be found in the BTBR mouse model of idiopathic autism.

## Materials and methods

### Human study

#### Participants

Thirty Japanese patients with ASD (21 males and 9 females, mean age = 29.8  ±  8.1 years) and 27 controls (19 males and 8 females, mean age = 30.3  ±  7.8 years) participated in this study (Table 1). All patients met the DSM-5 [1] criteria for ASD and were re-evaluated using the Autism Disorder Observation Schedule-2nd Edition (ADOS-2) [27]. Control group participants did not have a history of psychiatric, neurological, or developmental disorders, and were asked to complete the Mini-International Neuropsychiatric Interview to exclude their current or past psychiatric history. Moreover, we evaluated them using the Autism-Spectrum Quotient Japanese version (AQ-J) [28], and a score of < 27 was an enrollment requirement. Exclusion criteria for both groups included any neurological disorder, head injury, serious medical condition, or history of substance abuse or dependence. This study was approved by the Institutional Review Board of Nara Medical University and Sumitomo Pharma and conducted in accordance with the Declaration of Helsinki. Written informed consent was obtained from all participants prior to participation in the study. Sample size estimates were based on previous literature. No randomization was applied. No blinding was performed.

**Table 1:** Participant characteristics.

	Control		Patients with ASD		*t*-value	*p*-value
	*N* = 27		*N* = 30			
	Mean	SD	Mean	SD		
Sex (male/female)^a^	19/8		21/9		NA	1
Age (years)	30.3	7.8	28.8	7.1	0.75	0.46
Estimated IQ	108.6	10.0	99.8	12.4	2.97	4.4 × 10^−3^
Education (years)	15.9	2.2	14.1	2.2	3.12	2.9 × 10^−3^
AQ-J (total)	16.9	4.4	33.4	6.5	-11.4	1.1 × 10^−15^

^a^The χ^2^ test was used for testing group differences. Otherwise, two-sided Welch *t*-tests were used.

*AQ-J*, Autism-Spectrum Quotient Japanese version; *ASD*, autism spectrum disorder; *NA*, not applicable; *SD*, standard deviation.

#### Clinical measures

Clinical questionnaires including the Japanese version of Adolescent and Adult Sensory Profile (AASP) [29, 30] and AQ-J [28] were completed for all participants. ADOS-2 was assessed specifically for ASD patients. The Japanese version of Social Responsiveness Scale (SRS-2) [31] was assessed for both control and ASD groups, although not all participants could be evaluated. The intelligence quotient (IQ) of all participants was assessed with the Wechsler Adult Intelligence Scale-Fourth Edition (WAIS-IV).

#### ERP tasks

The ERP task was designed based on a previous study of auditory habituation in individuals with Fragile X Syndrome (FXS) [14]. The number of stimuli per train was set to four, as prior auditory ERP studies suggest that the majority of habituation effects typically occur within the first four presentations [14, 32]. A total of 150 trials were conducted with each trial consisting of a train of four 1000 Hz tones or fearful vocalizations (500-ms duration). Fearful vocalizations were selected from the Montreal Affective Voices database, specifically using the file 46_fear.wav [32], which was identified through a preliminary evaluation using visual analog scale (VAS) ratings as the stimulus that elicited the greatest difference between control and ASD groups. Stimuli within each train were separated by a 500-ms inter-stimulus interval; trials were separated by a 4000-ms interval. In passive listening tasks, presenting stimuli at relatively short intervals, such as within 500-ms, has been suggested to reduce cognitive influences on habituation [14]. All sounds were normalized to have the same root mean square (RMS) amplitude over the 500-ms duration. Stimuli were delivered at 70 db through headphones, while participants underwent dense-array electroencephalography (EEG).

#### Recording and analysis

ERPs were recorded using an EEG-1260 system (Nihon Kohden Corporation, Tokyo, Japan). EEGs were recorded over 64 Ag/AgCl electrodes placed according to the enhanced 10–20 system and arrayed in an elastic cap (Wavegard Cap). A reference electrode was placed at C3 and C4, and a ground electrode was placed at FPz. EEG recordings were pre-processed using Python’s MNE 1.8.0 package [33]. The completely automated preprocessing pipeline of the EEG signals was as follows. First, continuous EEG signals were high-passed filtered (>1 Hz), notch filtered at 60 Hz, and epoched with a time window of – 0.5 to 4.0 s relative to the first stimulus onset. Then noisy epochs were automatically rejected using Autoreject MNE packages [34], and electrooculogram (EOG) artefacts were detected via independent component analysis by using the Python Picard package. ICA components that are highly correlated with noise (>0.50, using the MNE component correlation method) in the Fpz channel were marked for later rejection. Raw data were reloaded, high-passed filtered (>1 Hz), notch filtered at 60 Hz, and epoched with a time window of – 0.5 to 4.0 s relative to the first stimulus onset, and EOG artefacts that were detected in previous steps were excluded. Epochs without EOG artefacts were applied automatic detection, repairing and rejection of noisy epochs by using Autoreject MNE packages. All subjects retained at least 67% of trials in the final analyses. Finally, the artefact-free epoch data were average re-referenced and low-pass filtered (<50 Hz), and event-related potentials (ERPs) were computed as the average of all the trials for each condition. We selected the frontal (Fz, F1, F2, FCz, FC1, and FC2), left parieto-occipital (P5, P7, PO3, PO5, and PO7), and right parieto-occipital (P6, P8, PO4, PO6, and PO8) ROIs for analysis, as these areas exhibited prominent P1 and N1 peaks (Figures S1 and S2). The P1 peaks were identified as the minimum value within the range of 10-80 ms from the onset of each stimulus (maximum value for the frontal region), and N1 peaks were identified as the maximum value within the range of 60–140 ms (minimum value for the frontal region). In the present study, the peak-to-peak amplitude (P1-N1 amplitude) was used to evaluate the magnitude of the response. This procedure minimizes issues associated with a baseline shift [35]. In a previous study of auditory habituation in individuals with FXS, the response to the fourth auditory stimulus was used as an approximation of the asymptote, based on the observation that most habituation occurred by the fourth presentation [14]. Following this approach, we quantified the difference in P1–N1 peak-to-peak amplitude between the first and fourth stimuli as an index of the degree of habituation in the present study.

### Mouse study

#### Animals

Fourteen C57BL/6J (Japan SLC, Inc.) and fourteen BTBR mice (BTBR T + tf/J, The Jackson Laboratory) were used in the present study. In addition, eighteen hemizygous male Fmr1 KO mice (B6.129P2-Fmr1tm1Cgr/J, The Jackson Laboratory) and nine wild-type littermates were also used. All animals were housed in a controlled environment (12-h light/dark cycle), with ad libitum access to diet and water. All experimental procedures involving mice were performed with the approval of Sumitomo Pharma Co., Ltd. Sample size estimates were based on previous literature. No randomization was applied. No blinding was performed.

#### Surgical procedure

Prefabricated EEG head mounts (8201, Pinnacle Technology, Inc.) were implanted in 9-week-old male mice. Mice were anesthetized with isoflurane and positioned on a stereotaxic frame using ear bars. A rostrocaudal midline incision was made in the skin to expose the skull surface. The head mount was positioned such that its posterior edge aligned with the lambda and its center aligned with the midline. Four pilot holes were tapped through the skull to the dura mater at positions corresponding to the screw holes of the head mount. Stainless steel screws were threaded into the pilot holes to anchor the head mount and simultaneously serve as EEG electrodes (EEG1 and EEG2), as well as reference and ground electrodes, targeting the frontal and parietal regions. The entire implant was insulated using dental acrylic. Mice were allowed to recover in their home cages for at least 4 days before EEG monitoring.

#### ERP tasks

Acoustic stimuli were generated using a TDT 3 and delivered to the animals via Multi-Field Magnetic Speakers (MF1; Tucker-Davis Technologies Inc.) located in the ceiling of the test chamber. There were 150 stimulus trials; each trial consisted of a train of four 20,000 Hz tones (50-ms duration). Tones within each train were separated by a 500-ms inter-stimulus interval; trials were separated by a 4000-ms interval. This stimulus paradigm was based on the tone condition used in the human experiment, which showed significant group differences and correlations with sensory hypersensitivity scores. The tone frequency was set to 20,000 Hz to match the auditory range of mice.

#### Recording and analysis

All experiments were carried out using EEG/EMG recording equipment (8400-K1; Pinnacle Technology Inc.) in sound-attenuating boxes. Auditory ERP recordings were obtained from freely moving mice and signals were amplified with a Pinnacle high-gain preamplifier (X100 amplification, 0.5 Hz high-pass filter and 100 Hz low-pass filter for EEG, and sampling rate, 400 Hz). Animals with severe movement artifacts or poor EEG waveforms were excluded. Original data were converted into a European data format (.edf) and analyzed using the Python MNE 1.8.0 package [33]. Continuous EEG signals were epoched with a time window of – 0.5 to 2.0 s relative to the first stimulus onset, and ERPs were computed as the average of all the trials. In this study, we analyzed data from EEG2, which represents recordings from the right anterior to the parieto-occipital region. Previous studies have reported that although auditory ERP components are conserved between rodents and humans, rodents exhibit shorter latencies [36]. For instance, the P1 component typically emerged at around 50 ms in humans and approximately 20 ms in mice. Similarly, the N1 component appeared at about 100 ms in humans and around 40 ms in mice. Based on these findings, the P1 peaks were identified as the maximum value within the range of 10-30 ms from the onset of each stimulus, and N1 peaks were identified as the minimum value within the range of 30-50 ms. The peak-to-peak amplitude (P1-N1 amplitude) was used to evaluate the magnitude of the response.

### Statistical analyses

We used R (version 4.4.0; R Core Team, 2024) for the statistical analyses. To compare the characteristics of participants in each group, two-sided Welch *t*-tests were used for continuous variables and Chi-square tests were used for discrete variables. Significant differences in IQ and education were observed between the control and ASD patients. Therefore, we performed a repeated-measures analysis of covariance (ANCOVA), using IQ and education as covariates, group (controls and ASD patients) as a between-subject factor, and ROIs (frontal, left parieto-occipital, and right parieto-occipital) as a within-subject factor. The Greenhouse-Geisser correction was applied in cases where the assumption of sphericity was violated. When significant group differences were observed using a repeated measures ANCOVA, we examined the group differences for each ROI using two-sided Welch *t*-tests with Bonferroni correction for multiple comparisons. Spearman correlation coefficients (rho) were calculated for the relationships between P1-N1 habituation responded to tones in the left parieto-occipital ROI and AASP sub-scores, AQ total score, ADOS-2 score, and SRS-2 score in the two groups. Clinical correlations were reported as part of exploratory analyses and were not corrected for multiple comparisons. In a mouse study, t-tests were used to compare each ERP component separately. A *p*-value of 0.05 was considered statistically significant.

## Results

### Demographic data

Demographic characteristics are presented in Table 1. There were no significant differences in sex (χ^2^  =  3.16 × 10^−31^, *df* = 1, *p* = 1.0) or age (*t*[53] = 0.75, *p* = 0.46) between the participant groups. However, there were significant between-group differences in average IQ (*t*[54] = 2.97, *p* = 0.0044) and education level (*t*[55] = 3.12, *p* = 0.0029).

### Comparison of P1-N1 peak-to-peak amplitudes to tones

**Fig. 1:**
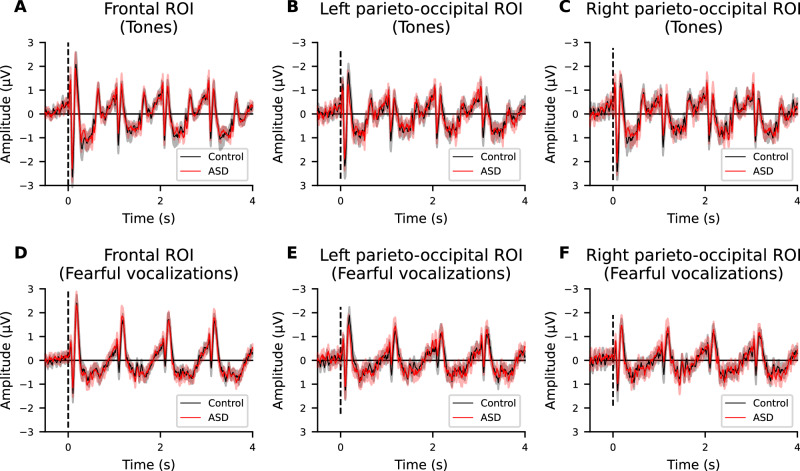
Event-related potential grand average in control individuals and patients with autism spectrum disorder (ASD). Grand averages for tones (**A**–**C**) and fearful vocalizations (**D**–**F**) in the control and ASD groups at the frontal (**A,** **D**), left parieto-occipital (**B,** **E**), and right parieto-occipital (**C,** **F**) regions of interest. The dashed line indicates the onset timing of the first auditory stimulus. The shaded areas in gray and red represent the mean and the 95% confidence interval.

**Fig. 2:**
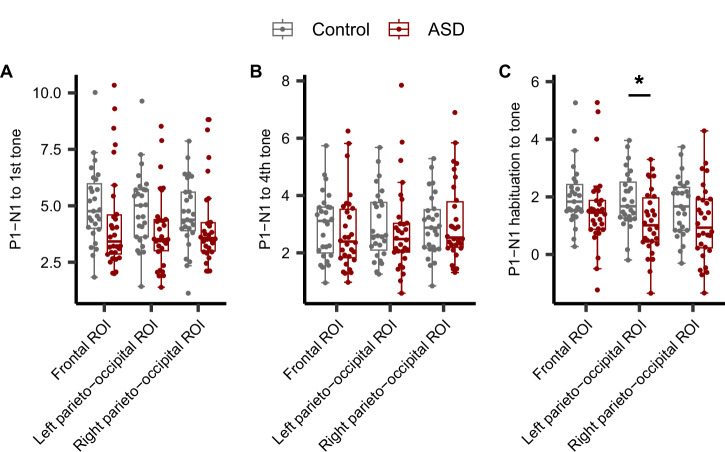
Quantification of the P1-N1 amplitude and habituation to tones. Group comparison of responses to the first tone (**A**), the fourth tone (**B**), and the degree of habituation (**C**) at the frontal, left parieto-occipital, and right parieto-occipital regions of interest. Box plots illustrate the first quartile, median, and third quartile, and 95% confidence limits. Circles show the individual data points. Black horizontal lines highlight significant differences between the groups (*p < 0.05, two-sided Welch *t*-tests with Bonferroni correction). The vertical axis is in microvolts (μV).

We defined three ROIs, the frontal ROI (Fz, F1, F2, FCz, FC1, and FC2), the left parieto-occipital ROI (P5, P7, PO3, PO5, and PO7) and the right parieto-occipital ROI (P6, P8, PO4, PO6, and PO8), where the P1 and N1 peaks were prominent for both tones and fearful vocalizations in both the control and ASD groups (Figures S1 and S2). Figure 1A–C shows the individual average waveforms for tones in each ROI (see also Figure S3 for separate plots of control and ASD groups). We compared the P1-N1 peak-to-peak amplitude in response to both the initial and fourth stimuli, and quantified their differences as indices of habituation. Controlling for IQ and education as covariates, we performed repeated measures ANCOVAs to determine whether the ERP measures differed between groups (Fig. 2). The repeated measures ANCOVA revealed a significant main effect of the group (*F* = 5.61, *p* =  0.022), while there were no significant interactions between group and ROI (*F* = 1.40, *p* =  0.25) on the P1-N1 peak-to-peak amplitude in response to the initial tone (Fig. 2A). For the P1-N1 peak-to-peak amplitude in response to the fourth tone, no significant group differences (*F* = 1.01, *p*  = 0.32) or interactions were observed (*F* = 1.53, *p* =  0.22) (Fig. 2B). The repeated measures ANCOVA for the degree of habituation also revealed a significant group difference (*F* = 7.51, *p* = 0.0084), but no significant interactions (*F* = 0.094, *p* =  0.88) (Fig. 2C). Next, we performed post-hoc tests for the effect of each ROI on the response to the first tonal stimulus and degree of habituation, where a significant intergroup difference was observed. As a result, it was revealed that only P1-N1 habituation in the left parieto-occipital ROI was significantly reduced in the ASD patient group compared to the control group (frontal ROI: response to the first tonal stimulus, *t*[54] = 1.37, *p* = 0.53; degree of habituation, *t*[54] = 1.54, *p* = 0.39; left parieto-occipital ROI: response to the first tonal stimulus, *t*[55] = 2.06, *p* = 0.13; degree of habituation, *t*[55] = 2.69, *p* = 0.028; right parieto-occipital ROI: response to the first tonal stimulus, *t*[54] = 0.97, *p* = 1.0; degree of habituation, *t*[54] = 1.85, *p* = 0.21; all p-values are Bonferroni corrected). These results support the notion that individuals with ASD exhibit reduced habituation to tones, consistent with findings from previous studies.

### Comparison of P1-N1 peak-to-peak amplitude to fearful vocalizations

**Fig. 3:**
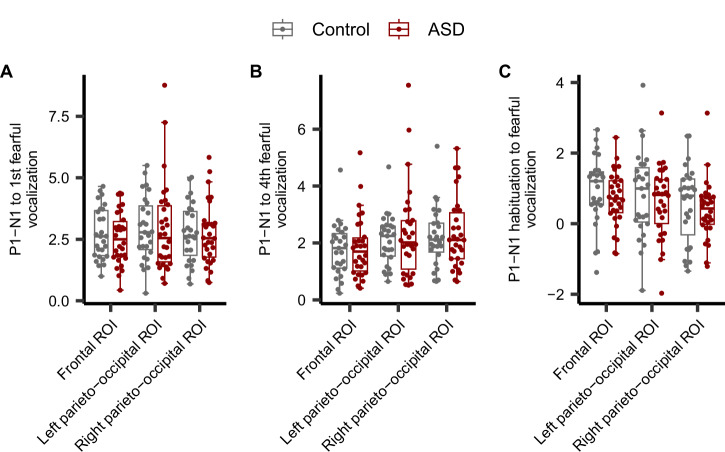
Quantification of the P1-N1 amplitude and habituation to fearful vocalizations. Group comparison of responses to the first fearful vocalization (**A**), the fourth fearful vocalization (**B**), and the degree of habituation (**C**) at the frontal, left parieto-occipital, and right parieto-occipital regions of interest. Box plots illustrate the first quartile, median, and third quartile, and 95% confidence limits. Circles show the individual data points. The vertical axis is in microvolts (μV).

Figure 1D–F shows the individual average waveforms for fearful vocalizations in each ROI (see also Figure S3 for separate plots of control and ASD groups). Similar to tonal stimuli, we also examined whether there were group differences in P1-N1 habituation to fearful vocalizations. We quantified the P1-N1 amplitude in response to the first and fourth stimulus, and the difference between the first and fourth P1-N1 amplitudes as the degree of habituation (Fig. 3). A repeated measures ANCOVA analysis controlling for IQ and education revealed no significant intergroup differences (*F* = 0.047, *p* = 0.83) or interactions between group and ROI (*F* = 0.071, *p* = 0.85) for the response to the first stimulus (Fig. 3A). Similarly, for the response to the fourth stimulus, no significant intergroup differences (*F* = 0.077, *p* = 0.78) or interactions between group and ROI (*F* = 0.080, *p* = 0.87) were observed (Fig. 3B). Furthermore, the degree of habituation, defined as the difference between the first and fourth P1-N1 amplitudes, also showed no significant intergroup differences (F = 0.43, p = 0.52) or interactions between group and ROI (*F* = 0.022, *p* = 0.94) (Fig. 3C). Contrary to our expectations, there were no significant differences in the response to fearful vocalizations between control and ASD patients. These results suggest that individuals with ASD may exhibit more impaired habituation to simple stimuli like tones rather than to emotionally evocative stimuli.

### Correlation between ERP components and clinical measures

**Fig. 4:**
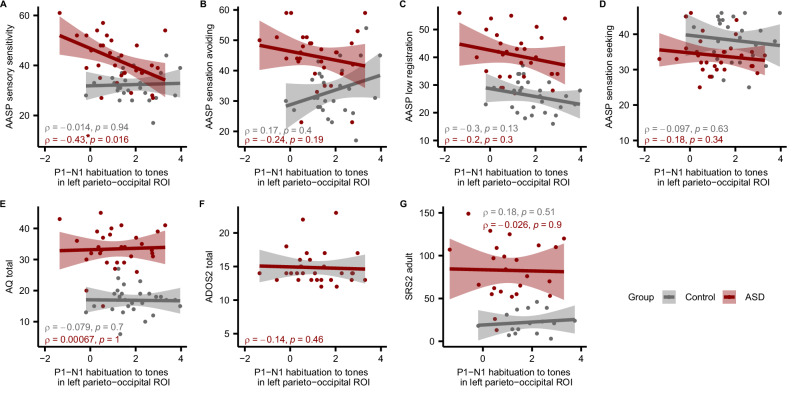
Correlations between P1-N1 habituation to tones in the left parieto-occipital region of interest (ROI) and clinical scores. Correlations between P1**-**N1 habituation to tonal stimuli in the left parieto-occipital ROI and Adolescent and Adult Sensory Profile (AASP) sensory sensitivity (**A**), AASP sensation avoiding (**B**), AASP low registration (**C**), AASP sensation seeking (**D**), Autism Spectrum Quotient total (**E**), Autism Disorder Observation Schedule-2nd Edition total (**F**), and Social Responsiveness Scale-2 adult (**G**). Lines represent the regression lines for each group, shaded areas indicate the confidence intervals, and circles represent individual samples. The Spearman rho coefficients (ρ) and uncorrected *p*-values are indicated.

Next, we examined whether the P1-N1 habituation responses to tones in the left parieto-occipital ROI could serve as a biomarker reflecting sensory hypersensitivity symptoms. This region showed a significant group difference between the control and ASD patient groups. We investigated the correlation with AASP sub-scores. In the ASD patients, a moderate significant correlation was found between the P1-N1 habituation responses to tones in the left parieto-occipital ROI and the AASP sensory sensitivity sub-score (ρ = -0.43 *p* = 0.016) (Fig. 4A). On the other hand, for other AASP sub-scores, the correlations with P1-N1 habituation to tones in the left parieto-occipital ROI in ASD patients were weak and not significant (Fig. 4B–D). We further examined the correlation with the severity of autism. In ASD patients, no significant correlations were found between the P1-N1 habituation to tones in the left parieto-occipital ROI and the AQ total score, ADOS-2 score, or SRS-2 score (Fig. 4E–G). These results suggest that P1-N1 habituation to tones in the left parieto-occipital ROI may be particularly associated with sensory hypersensitivity symptoms in ASD patients, and the use of such biomarkers may be useful for the objective diagnosis of sensory hypersensitivity symptoms and assessment of treatment efficacy.

### Back-translation into rodent models

**Fig. 5:**
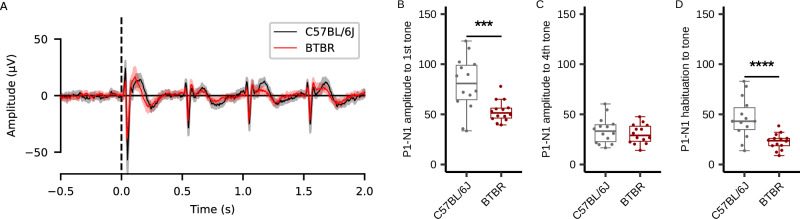
Event-related potential grand average, quantification of the P1-N1 amplitude and habituation of C57BL/6J and BTBR mice. **A** Grand average waveforms for C57BL/6J and BTBR mice. The dashed line indicates the onset timing of the first auditory stimulus. The shaded areas in gray and red represent the mean and the 95% confidence interval. (B-D) Quantitative results: responses to the first stimulus (**B**), responses to the fourth stimulus (**C**), and the degree of habituation (**D**). Box plots illustrate the first quartile, median, and third quartile, and 95% confidence limits. Circles show the individual data points. Black horizontal lines highlight significant differences between the groups (***p < 0.005, ****p < 0.001, two-sided Welch *t*-tests). The vertical axis is in microvolts (μV). Each group consisted of 14 mice (n = 14 per group).

Finally, we examined the translational potential of the biomarker, reduced P1-N1 habituation to tones, observed in ASD patients. We presented four tonal stimuli to control (C57BL6/J) mice and BTBR mice, which are widely used as ASD model mice, and analyzed the event-related potentials. Figure 5A shows the grand average waveforms for control and BTBR mice in response to the four tonal stimuli (see also Figure S4 for separate plots of control and BTBR). We compared the P1-N1 peak-to-peak amplitude in response to both the initial and fourth stimuli, and quantified their differences as indices of habituation. We found that BTBR mice, compared to control mice, showed a significantly reduced P1-N1 peak-to-peak amplitude in response to the first stimulus and a lower degree of habituation (response to the first stimulus: *t*[17] = 3.45, *p* = 0.0031; response to the fourth stimulus: *t*[24] = 0.81, *p* = 0.43; degree of habituation: *t*[17] = 3.98, *p* = 0.00098) (Fig. 5B–D). This result was consistent with findings from clinical studies comparing control to ASD groups. To examine whether similar abnormalities of ERP habituation could be observed in other ASD model mice under the same experimental condition, we tested Fmr1 knockout (KO) mice, a well-established model of FXS, which is the most common monogenetic form of ASD. Figure S5A shows the grand average waveforms for wild-type littermates (WT) and Fmr1 KO mice in response to the four tonal stimuli (see also Figure S5E and S5F for separate plots of WT and Fmr1 KO). Although all measures showed a trend toward reduction in Fmr1 KO mice compared to WT, these differences did not reach statistical significance (response to the first stimulus: *t*[10] = 1.97, *p* = 0.076; response to the fourth stimulus: *t*[10] = 2.11, *p* = 0.062; degree of habituation: *t*[10] = 1.43, *p* = 0.19) (Figure S5B–S5D). Therefore, under the experimental conditions used in this study, BTBR mice appear to more closely resemble the ERP habituation abnormalities observed in idiopathic ASD patients.

## Discussion

Sensory hypersensitivity is a symptom with substantial impact on ASD patients and their caregivers, and understanding its pathophysiological mechanisms is crucial. One of the causes of sensory hypersensitivity is reported to be deficits in sensory filtering functions, such as sensory habituation and gating, in response to repeated stimuli. Additionally, sensory hypersensitivity is closely related to anxiety, with some theories suggesting that it arises as a secondary effect of anxiety [19]. Indeed, several studies have suggested that emotions can influence sensory filtering functions [20–22]. However, previous studies on habituation and gating in ASD patients have primarily used stimuli such as tones or clicks, and there has been little research examining abnormalities in habituation to emotionally charged stimuli [7–16]. Our study is the first to compare the degree of habituation to tones and fearful vocalizations in both neurotypical controls and ASD patients using the same paradigm and the same subjects. We found that habituation to tones was significantly reduced in the ASD group, whereas there was no significant difference between groups in habituation to fearful vocalizations. These results can be interpreted in two major ways. First, it is possible that ASD patients have an impairment of sensory filtering functions, which is why there was a significant difference between groups for tones, stimuli that typically elicit habituation in controls, but not for fearful vocalizations, stimuli that are less likely to be filtered even in controls. Second, it is possible that ASD patients experience anxiety even in response to tones, resulting in decreased sensory filtering of tonal stimuli. Recent studies have supported the notion that sensory issues arise earlier and are primary in ASD, with anxiety developing as a secondary consequence. For example, it has been reported that sensory hypersensitivity symptoms can predict increased anxiety symptoms, but not vice versa [37], and that hyperactive brain responses to aversive sensory stimulation are more highly related to sensory hypersensitivity symptoms than anxiety symptoms in ASD [38]. Therefore, we hypothesize that the former explanation, that ASD patients have an impairment in sensory filtering function, is more likely, although further research is needed. The specific correlation found in this study between P1-N1 habituation to tones and clinical scores of sensory hypersensitivities also supports the idea that abnormalities in sensory filtering functions are a cause of sensory hypersensitivity symptoms in ASD patients.

Sensory hypersensitivity is observed not only in ASD but also across various neurodevelopmental and psychiatric disorders [39–43]. Research on biomarkers for sensory hypersensitivity symptoms has been particularly active in patients with Fragile X Syndrome (FXS) [44, 45]. It has been reported that in patients with FXS, the N1 amplitude in response to auditory stimuli is increased, whereas in patients with ASD, the N1 amplitude tends to decrease, suggesting that there may be differences in how sensory processing is impaired in the two disorders [46, 47]. In this study, a similar trend was observed, with the P1-N1 amplitudes in response to the first stimulus being reduced in ASD patients, consistent with previous research findings. On the other hand, in terms of habituation to repeated auditory stimuli, the degree of habituation was reduced in ASD patients compared to the control group, similar to FXS patients, and this reduction correlated with clinical scores of sensory hypersensitivity symptoms. This suggests that abnormalities in habituation may serve as a biomarker for sensory hypersensitivity symptoms common to both FXS and ASD. However, it is important to recognize that the relationship between habituation and sensory hypersensitivity may not be consistent across all disorders; for instance, in Phelan-McDermid Syndrome (PMS), greater initial N1 amplitude and greater habituation were associated with higher scores of auditory hypersensitivities [48]. Future studies should investigate whether such habituation abnormalities broadly contribute to sensory hypersensitivity across different disorders.

In the development of drugs for psychiatric and neurodevelopmental disorders, there is a need to develop translational biomarkers associated with specific symptom domains [49]. The translational potential of biomarkers related to sensory hypersensitivity has been primarily investigated using genetic ASD models such as a mouse model of FXS [25, 26, 50]. Given that the majority of ASD cases are idiopathic, it remains largely unexplored whether neurophysiological abnormalities observed in idiopathic ASD patients can be recapitulated in idiopathic animal models. In this study, we investigated whether the biomarker of P1-N1 habituation could be back-translated to preclinical settings using BTBR mice. BTBR mice are widely used as an animal model of ASD and have been reported to exhibit sensory processing abnormalities similar to those of ASD patients [51–53]. Furthermore, it has already been reported that the hearing ability of BTBR is comparable to that of other mouse strains [54]. Nevertheless no ERP studies have been conducted to date, and prior behavioral assessments such as prepulse inhibition have not identified sensorimotor gating deficits in BTBR mice [55]. To our knowledge, this is the first study to examine ERP habituation—a neurophysiological index—in BTBR mice, and to demonstrate that these animals exhibit habituation deficits analogous to those found in ASD patients. In addition to BTBR mice, we also examined the Fmr1 knockout (Fmr1KO) mice, a widely used model of FXS. Structural and functional neuroimaging studies of ASD models have categorized BTBR and Fmr1 KO mice into distinct clusters [56, 57], indicating that these models may represent different subtypes within ASD. Although previous studies have reported increased N1 amplitudes and reduced N1 habituation in Fmr1 KO mice [25, 58, 59], neither finding was replicated in the present study. It has been suggested that these abnormalities might only be present under specific experimental conditions [26, 50], which may underlie the discrepancies observed in our results. To determine whether auditory habituation deficits are broadly shared across distinct subtypes of ASD mouse models, future studies should systematically compare habituation responses across multiple models. Such comparative approaches will be essential for clarifying the extent to which habituation deficits reflect a shared neurophysiological signature of ASD. Furthermore, it remains uncertain whether the mechanisms underlying auditory habituation deficits in BTBR mice are truly shared with those in ASD patients. Future research aimed at elucidating the mechanisms of auditory habituation in both BTBR mice and ASD individuals, and identifying ASD subtypes that share pathophysiological features with BTBR mice, may pave the way for more targeted and effective therapeutic strategies.

In this study, although habituation tends to decrease similarly in the frontal, left, and right parieto-occipital region, a statistically significant difference was observed only in the left parieto-occipital region. The tendency for left-dominant abnormalities in ASD patients has been reported in several previous studies. For example, multiple meta-analyses have reported structural brain abnormalities, such as abnormalities in language-associated fibers, that are particularly pronounced in the left hemisphere of ASD patients [60, 61]. Additionally, neurophysiological studies have also reported left-hemispheric abnormalities, such as shortened latency of mismatch negativity in response to novel vowel sounds in ASD patients with language impairments [62], and a left-hemispheric sustained field deficit in response to 40-Hz click trains in children with ASD [63]. Furthermore, although there are few reports on the relationship with sensory hypersensitivity symptoms, a study on children born with very low birth weight reported a correlation between voice-evoked responses in the left hemisphere and sensory hypersensitivity symptoms [64]. Our results are consistent with these previous findings, suggesting that sensory processing issues in the left hemisphere may underlie sensory hypersensitivity symptoms in ASD.

This study has several limitations. First, there were differences in IQ and educational background between the groups, which, despite statistical adjustments, suggest that comparisons between more closely matched groups would be preferable. Additionally, this study focused on adults. While sensory issues are widely recognized in adult ASD patients, they are said to be most pronounced in children, necessitating future studies focusing on children. Another limitation is related to the evaluation of responses to repeated stimuli. Specifically, baseline shift poses a significant issue in such studies. In this study, we adopted a method of quantifying the P1-N1 peak-to-peak amplitude without baseline removal. This method minimizes the impact of baseline shift and achieves a high signal-to-noise ratio [35, 65]. While evaluating P1-N1 simplifies the results and makes interpretation easier, it should be noted that there may be different effects on each peak, P1 and N1, that have not been considered. Another limitation of this study pertains to its translational design. In this study, the mouse experiment was designed based on a human experimental paradigm; however, the optimal conditions for inducing habituation may vary across species. Indeed, a recent study has suggested that the duration of stimulus required to elicit suppressive neural activity associated with adaptation may differ by species [66]. These species-specific differences highlight the importance of investigating the underlying mechanisms of auditory habituation abnormalities in both humans and mice. Elucidating these mechanisms is crucial for establishing construct validity, which in turn is essential for ensuring the translational relevance of this biomarker. Finally, the sample size is not large, which is a concern given the heterogeneous nature of ASD. Conducting larger studies in the future could not only confirm the reproducibility of our findings but also potentially enable the stratification of ASD patients into different groups based on the underlying causes of their sensory issues using EEG and ERPs.

## Supplementary information

Supplemental Material

## Data Availability

The data that support the findings of this study are available from the corresponding author upon reasonable request.
