# Supplementary material for: Comparative study of ERP habituation to tones and fearful vocalizations in autism spectrum disorders: a translational biomarker for sensory hypersensitivity
Source: Mol Psychiatry. 2025 Nov 5;31(4):1871–9. doi: 10.1038/s41380-025-03335-z (PMC12999479; doi:10.1038/s41380-025-03335-z)
Supplement: Supplementary file 1 — Supplemental Material [file 41380_2025_3335_MOESM1_ESM.pdf]

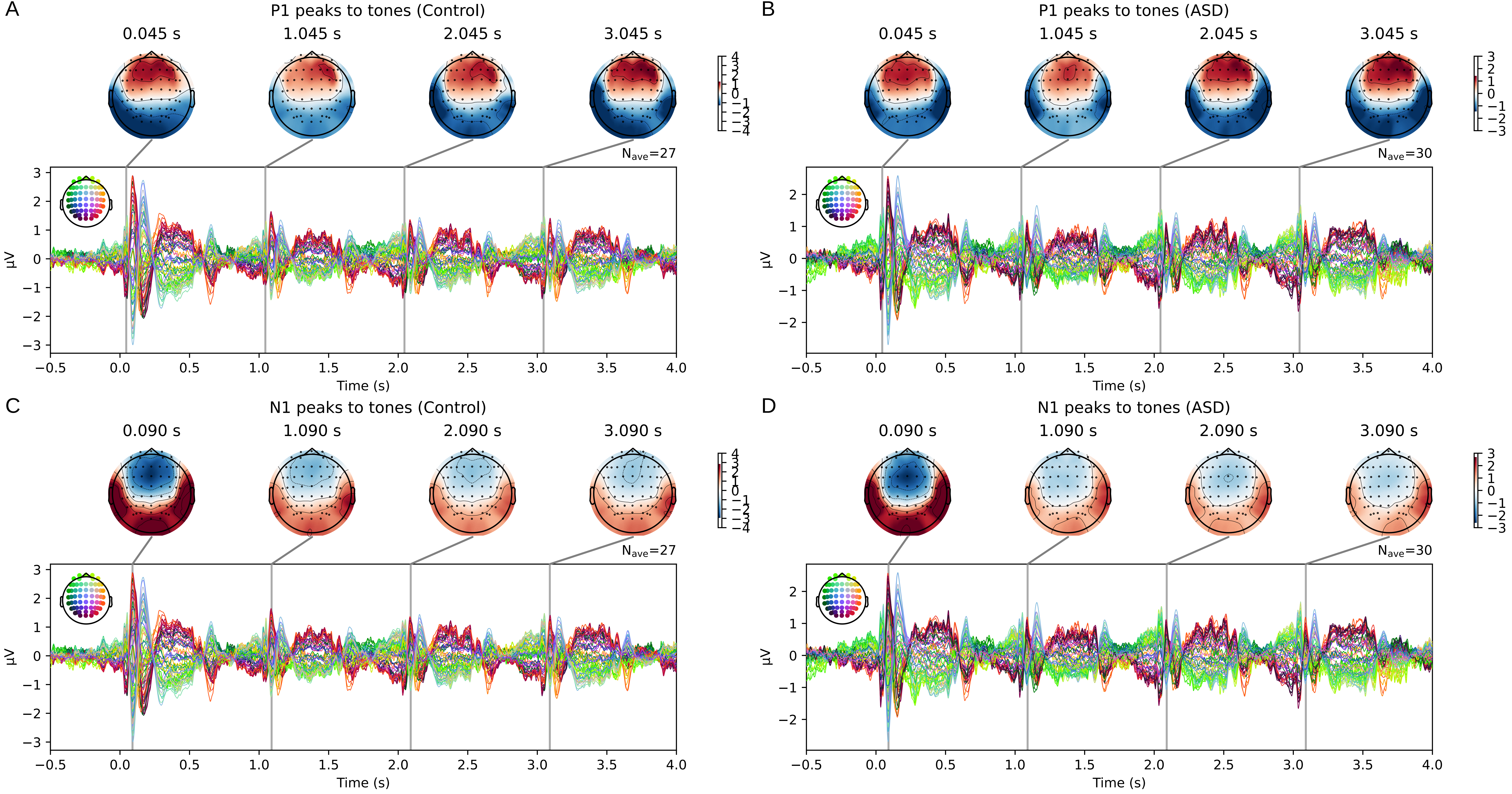

A
P1 peaks to tones (Control)
0.045 s
1.045 s
2.045 s
3.045 s
Nave=27
µV
Time (s)
B
P1 peaks to tones (ASD)
0.045 s
1.045 s
2.045 s
3.045 s
Nave=30
µV
Time (s)
C
N1 peaks to tones (Control)
0.090 s
1.090 s
2.090 s
3.090 s
Nave=27
µV
Time (s)
D
N1 peaks to tones (ASD)
0.090 s
1.090 s
2.090 s
3.090 s
Nave=30
µV
Time (s)

**Figure S1. Grand average event-related potential waveforms and topographic maps of the P1 and N1 to tones**

Average electroencephalogram waveforms evoked by the stimulus are plotted for each of the 64 electrodes, with each channel color-coded. (A) and (C) show control data, while (B) and (D) show data from autism spectrum disorders participants. (A) and (B) display grand averages and topographic maps corresponding to the P1 peaks for each stimulus, and (C) and (D) display grand averages and topographic maps corresponding to the N1 peaks for each stimulus. Each figure includes the grand average of four tonal stimuli, with topographic maps at specific timings. Frontal electrodes show positive activity at times corresponding to the P1 response and negative activity at times corresponding to the N1 response, whereas occipital and parietal electrodes show negative activity at times corresponding to the P1 response and positive activity at times corresponding to the N1 response.

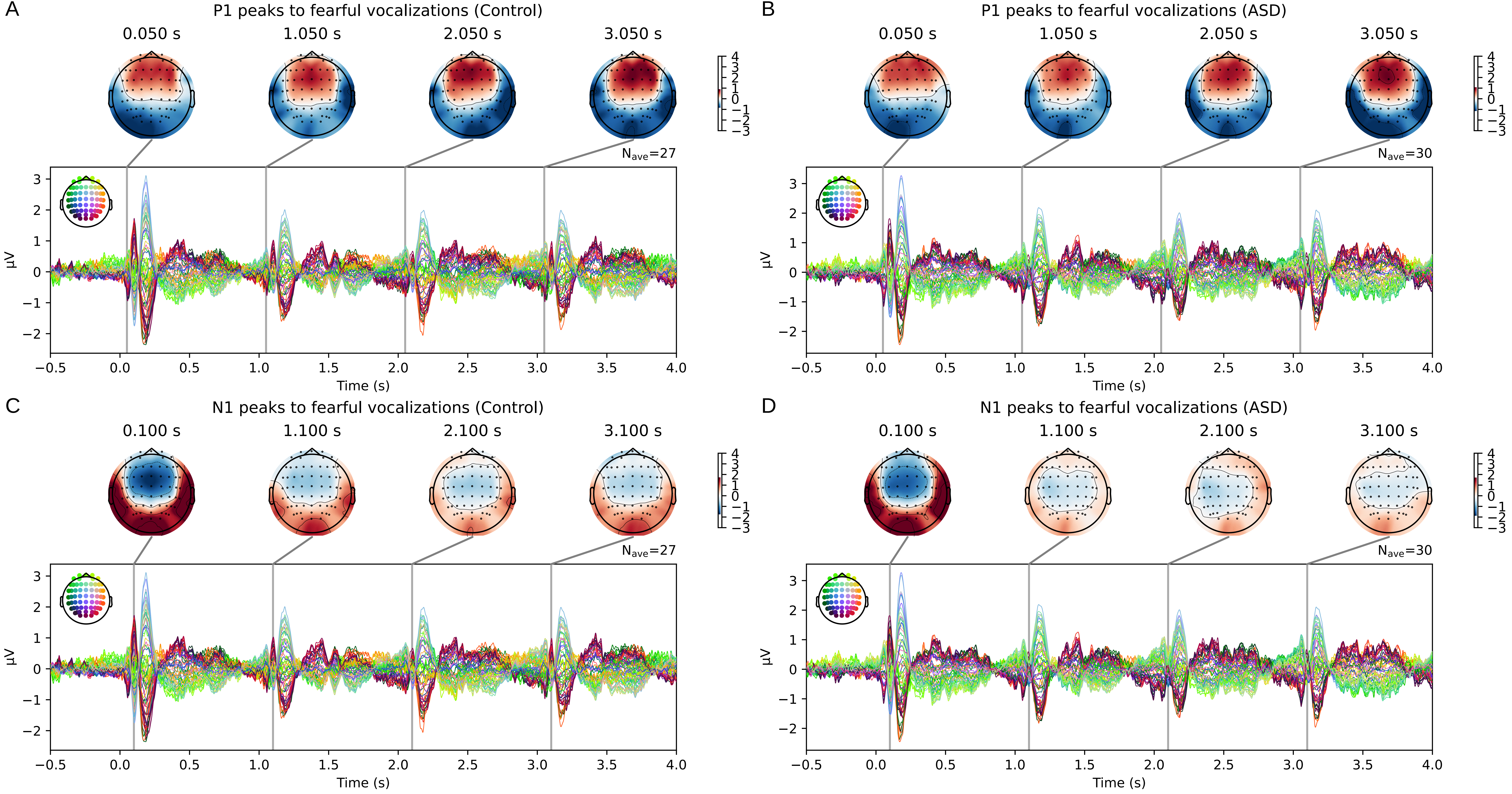
A
P1 peaks to fearful vocalizations (Control)
0.050 s
1.050 s
2.050 s
3.050 s
N_ave=27
μV
Time (s)
B
P1 peaks to fearful vocalizations (ASD)
0.050 s
1.050 s
2.050 s
3.050 s
N_ave=30
μV
Time (s)
C
N1 peaks to fearful vocalizations (Control)
0.100 s
1.100 s
2.100 s
3.100 s
N_ave=27
μV
Time (s)
D
N1 peaks to fearful vocalizations (ASD)
0.100 s
1.100 s
2.100 s
3.100 s
N_ave=30
μV
Time (s)

**Figure S2. Grand average event-related potential waveforms and topographic maps of the P1 and N1 to fearful vocalizations**

Average electroencephalogram waveforms evoked by the stimulus are plotted for each of the 64 electrodes, with each channel color-coded. (A) and (C) show control data, while (B) and (D) show data from ASD participants. (A) and (B) display grand averages and topographic maps corresponding to the P1 peaks for each stimulus, and (C) and (D) display grand averages and topographic maps corresponding to the N1 peaks for each stimulus. Each figure includes the grand average of four tonal stimuli, with topographic maps at specific timings. Frontal electrodes show positive activity at times corresponding to the P1 response and negative activity at times corresponding to the N1 response, whereas occipital and parietal electrodes show negative activity at times corresponding to the P1 response and positive activity at times corresponding to the N1 response.

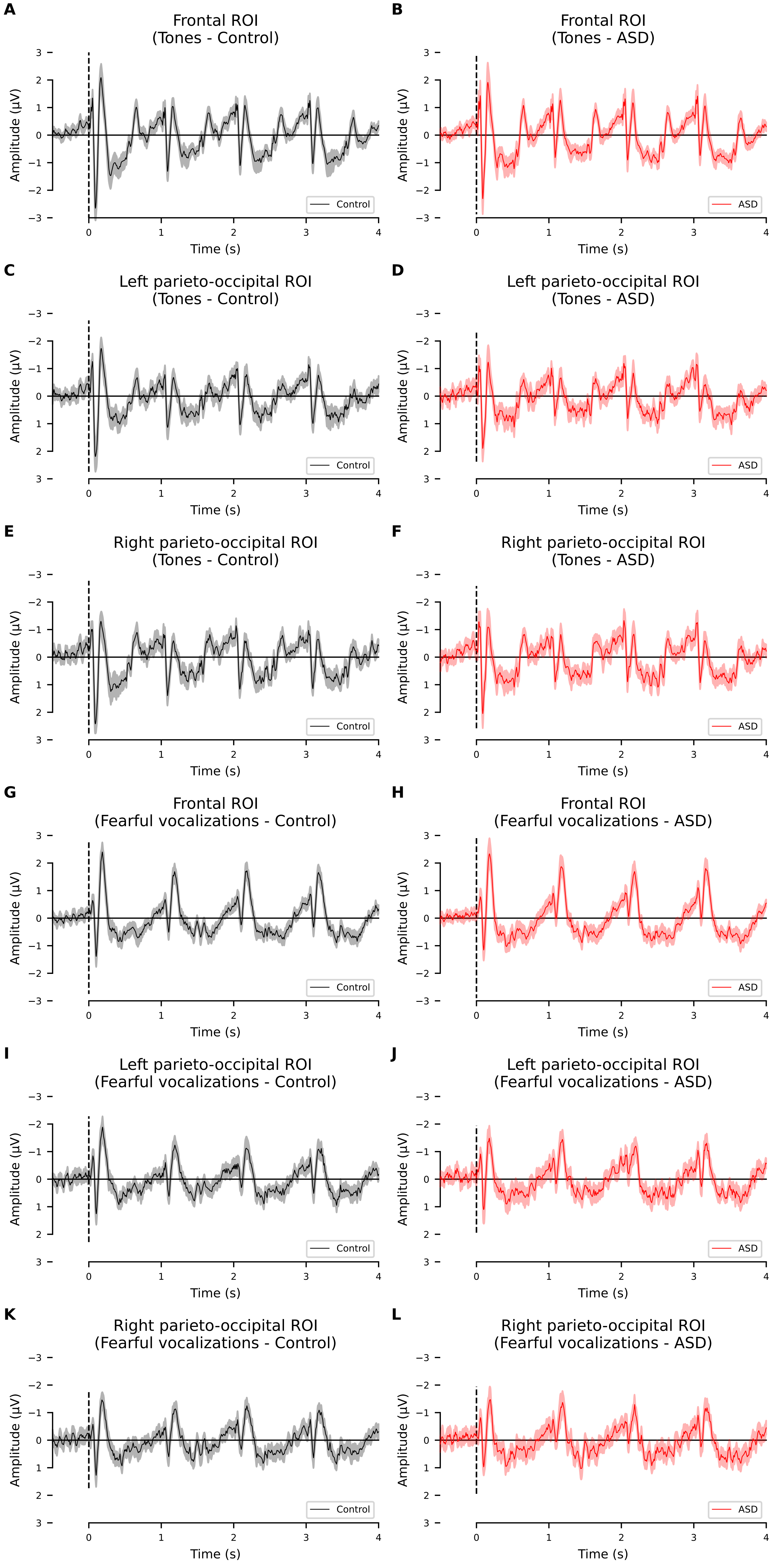
A
Frontal ROI
(Tones - Control)
B
Frontal ROI
(Tones - ASD)
C
Left parieto-occipital ROI
(Tones - Control)
D
Left parieto-occipital ROI
(Tones - ASD)
E
Right parieto-occipital ROI
(Tones - Control)
F
Right parieto-occipital ROI
(Tones - ASD)
G
Frontal ROI
(Fearful vocalizations - Control)
H
Frontal ROI
(Fearful vocalizations - ASD)
I
Left parieto-occipital ROI
(Fearful vocalizations - Control)
J
Left parieto-occipital ROI
(Fearful vocalizations - ASD)
K
Right parieto-occipital ROI
(Fearful vocalizations - Control)
L
Right parieto-occipital ROI
(Fearful vocalizations - ASD)
Amplitude (µV)
Time (s)
Control
ASD

**Figure S3. Event-related potential grand average in control individuals and patients with autism spectrum disorder (ASD)**

Grand averages for tones (A-F) and fearful vocalizations (G-L) in the control (A, C, E, G, I and K) and ASD groups (B, D, F, H, J and L) at the frontal (A, B, G and H), left parieto-occipital (C, D, I and J), and right parieto-occipital (E, F, K and L) regions of interest. The dashed line indicates the onset timing of the first auditory stimulus. The shaded areas in gray and red represent the mean and the 95% confidence interval.

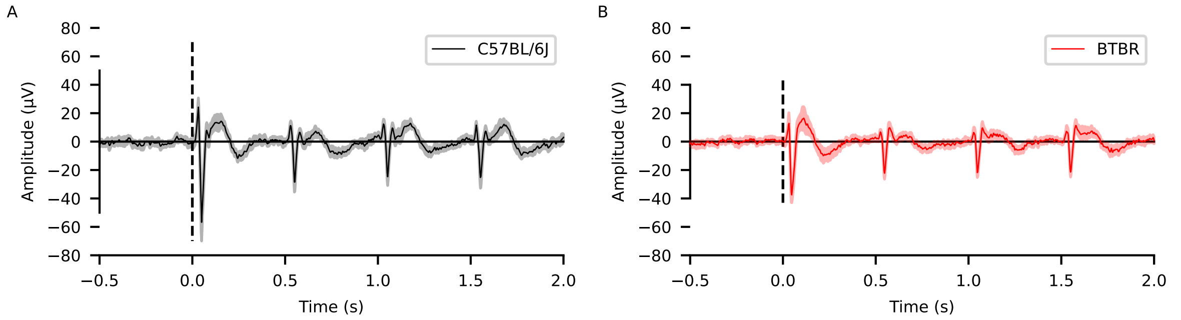
A
B
C57BL/6J
BTBR
Amplitude (µV)
Time (s)
80
60
40
20
0
−20
−40
−60
−80
−0.5
0.0
0.5
1.0
1.5
2.0

**Figure S4. Event-related potential grand average in C57BL/6J and BTBR mice**

Grand average waveforms for C57BL/6J (A) and BTBR mice (B). The dashed line indicates the onset timing of the first auditory stimulus. The shaded areas in gray and red represent the mean and the 95% confidence interval.

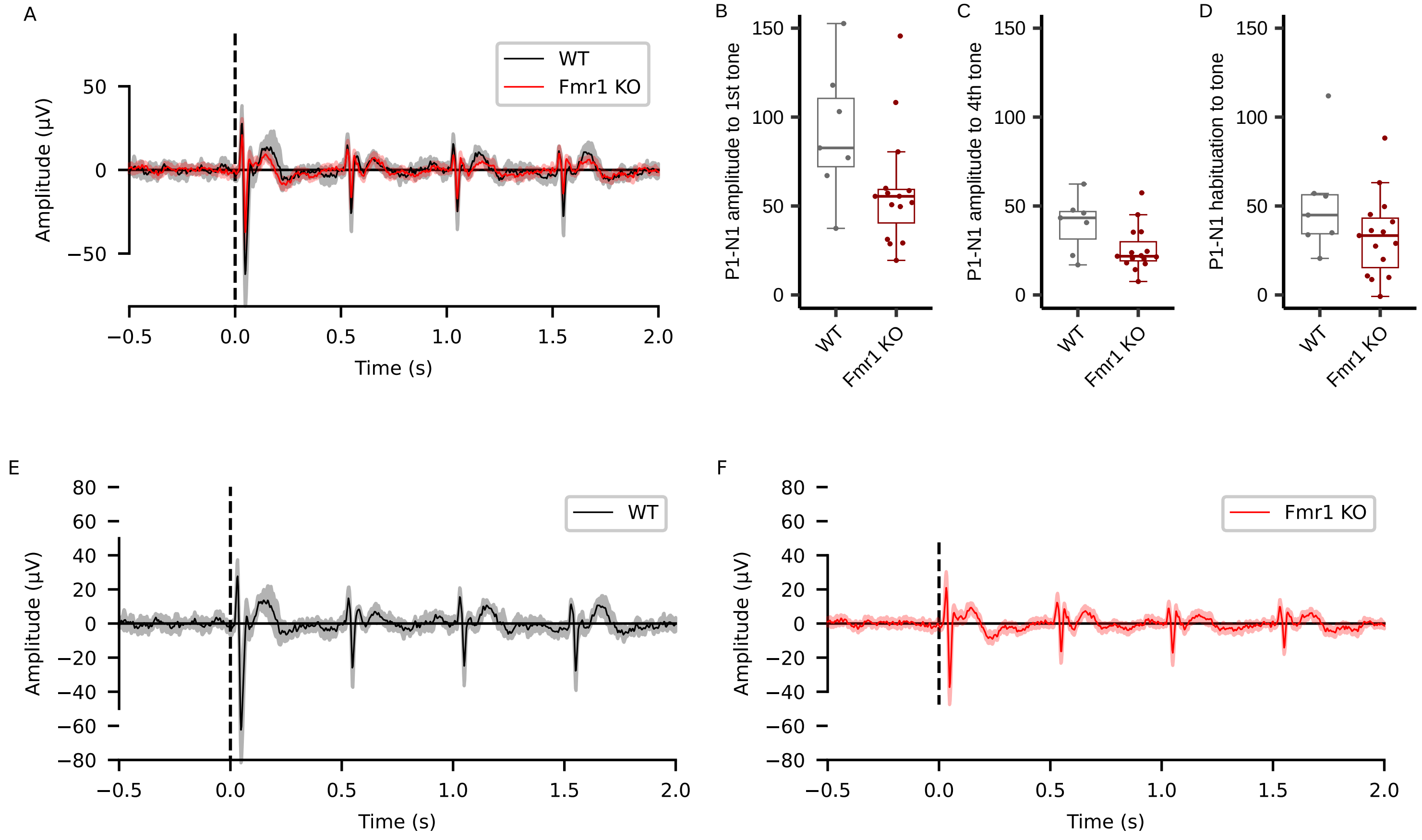
A
B
C
D
E
F
WT
Fmr1 KO
Amplitude (µV)
Time (s)
P1-N1 amplitude to 1st tone
P1-N1 amplitude to 4th tone
P1-N1 habituation to tone

**Figure S5. Event-related potential grand average, quantification of the P1-N1 amplitude and habituation of wild-type and Fmr1 KO mice**

(A) Grand average waveforms for Fmr1 KO mice compared to wild-type littermates (WT). (B-D) Quantitative results: responses to the first stimulus (B), responses to the fourth stimulus (C), and the degree of habituation (D). Box plots illustrate the first quartile, median, and third quartile, and 95% confidence limits. Circles show the individual data points. The vertical axis is in microvolts (μV). WT: n = 7; Fmr1 KO: n = 15. Grand average waveforms for WT (E) and Fmr1 KO mice (F). The dashed line indicates the onset timing of the first auditory stimulus. The shaded areas in gray and red represent the mean and the 95% confidence interval.
